# Supplementary material for: Identification of photoperiod-induced specific miRNAs in the adrenal glands of Sunite sheep (Ovis aries)
Source: Front Vet Sci. 2022 Jul 22;9:888207. doi: 10.3389/fvets.2022.888207 (PMC9354845; doi:10.3389/fvets.2022.888207)
Supplement: Supplementary file 1 [file Data_Sheet_1.ZIP › Supplementary file/miRDeep2 file.zip!/miRDeep2 file/Know_Structure/SPLP42A1/oar-mir-30a.pdf]

miRBase precursor : oar-mir-30a
Total read count : 91595
oar-miR-30a-5p read count : 85683
oar-miR-30a-3p read count : 5912
remaining reads : 0

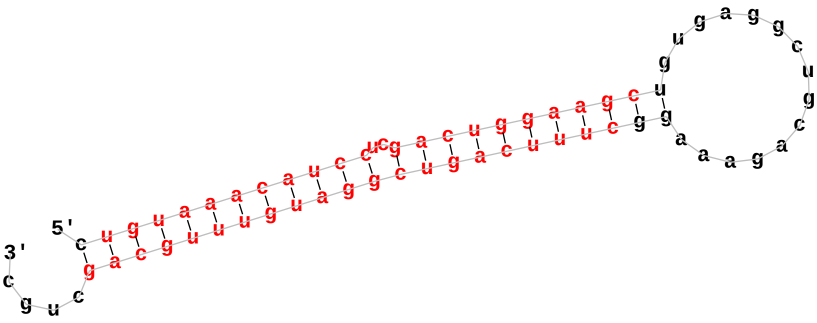

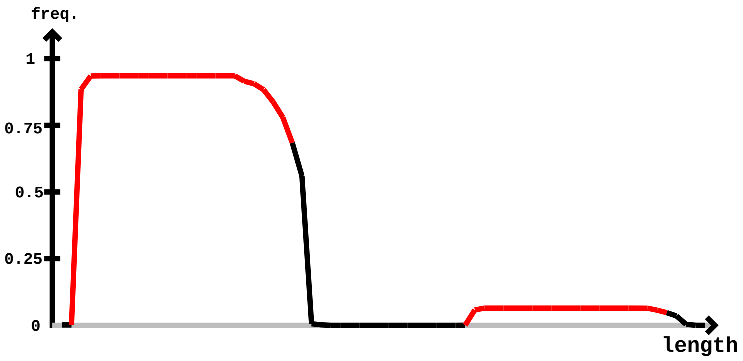

oar-miR-30a-3p

oar-miR-30a-5p

5'- cuguaaacauccucgacuggaagcugugaggcugcagaaaggcuuucagucggauguuugcagcugc -3'	exp		
((((((((((((..(((((((((((...............)))))))))))))))))))))))....	reads	mm	sample
cuguaaacauccuUgacu...	1	1	seq
cuguaaacauccucgacu...	1	0	seq
cuguaaacauccuUgacug..	1	1	seq
cuguaaacauccucgacugg...	5	0	seq
cuguaaacauccucgacugga..	18	0	seq
cuguaaacauccuUgacugga..	2	1	seq
cuguaaacauccucgacuggaa...	14	0	seq
cuguaaacauccuUgacuggaag..	1	1	seq
cuguaaacauccucgacuggaag..	9	0	seq
cuguaaacauccuUgacuggaagc...	4	1	seq
cuguaaacauccucgacuggaagc...	25	0	seq
cuguaaacauccucgacuggaagcu..	24	0	seq
cuguaaacauccuUgacuggaagcu..	24	1	seq
.uguaaacauccucgacu...	207	0	seq
.uguaaacauccuUgacu...	1652	1	seq
.uguaaacauccuUgacug..	646	1	seq
.uguaaacauccucgacug..	209	0	seq
.uguaaacauccucgacugg...	857	0	seq
.uguaaacauccuUgacugg...	1158	1	seq
.uguaaacauccucgacugga..	3400	0	seq
.uguaaacauccuUgacugga..	793	1	seq
.uguaaacauccucgacuggaa...	4399	0	seq
.uguaaacauccuUgacuggaa...	819	1	seq
.uguaaacauccuUgacuggaag..	2923	1	seq
.uguaaacauccucgacuggaag..	5851	0	seq
.uguaaacauccuUgacuggaagc...	1878	1	seq
.uguaaacauccucgacuggaagc...	9412	0	seq
.uguaaacauccucgacuggaagcu..	18571	0	seq
.uguaaacauccuUgacuggaagcu..	28020	1	seq
.uguaaacauccucgacuggaagcug...	23	0	seq
.uguaaacauccuUgacuggaagcug...	76	1	seq
.uguaaacauccucgacuggaagcugu..	5	0	seq
.uguaaacauccuUgacuggaagcugu..	15	1	seq
..guaaacauccuUgacugg...	2	1	seq

```
                                          oar-miR-30a-3p
 oar-miR-30a-5p
cuguaaacauccucgacuggaagcugugaggcugcagaaaggcuuucagucggauguuugcagcugc

..guaaacauccuUgacugga..............................................    7      1     seq
..guaaacauccucgacugga..............................................    3      0     seq
..guaaacauccucgacuggaa.............................................    3      0     seq
..guaaacauccuUgacuggaa.............................................    7      1     seq
..guaaacauccuUgacuggaag............................................    37     1     seq
..guaaacauccucgacuggaag............................................    6      0     seq
..guaaacauccucgacuggaagc...........................................    19     0     seq
..guaaacauccuUgacuggaagc...........................................    41     1     seq
..guaaacauccuUgacuggaagcu..........................................    3899   1     seq
..guaaacauccucgacuggaagcu..........................................    173    0     seq
..guaaacauccuUgacuggaagcug.........................................    255    1     seq
..guaaacauccucgacuggaagcug.........................................    2      0     seq
..guaaacauccuUgacuggaagcugu........................................    138    1     seq
...uaaacauccucgacuggaa.............................................    1      0     seq
...uaaacauccuUgacuggaag............................................    1      1     seq
...uaaacauccucgacuggaagc...........................................    1      0     seq
...uaaacauccuUgacuggaagcu..........................................    17     1     seq
...uaaacauccucgacuggaagcu..........................................    5      0     seq
...uaaacauccuUgacuggaagcug.........................................    13     1     seq
...uaaacauccucgacuggaagcug.........................................    1      0     seq
...uaaacauccuUgacuggaagcugu........................................    8      1     seq
....aaacauccuUgacuggaagcugu........................................    1      1     seq
.........................................gcuuucagucggauguuu........    1      0     seq
.........................................gcuuucagucggauguuug.......    1      0     seq
.........................................gcuuucagucggauguuuAc......    5      1     seq
.........................................gcuuucagucggauguuuAca.....    10     1     seq
.........................................gcuuucagucggauguuuAcag....    4      1     seq
.........................................gcuuucagucggauguuuAcagc...    2      1     seq
..........................................cuuucagucggauguuu........    10     0     seq
..........................................Auuucagucggauguuu........    1      1     seq
..........................................cuuucagucggauguuuA.......    16     1     seq
..........................................cuuucagucggauguuug.......    8      0     seq
..........................................cuuucagucggauguuugc......    125    0     seq
..........................................cuuucagucggauguuuAc......    507    1     seq
..........................................cuuucagucggauguuugca.....    83     0     seq
..........................................cuuucagucggauguuuAca.....    736    1     seq
..........................................cuuucagucggauguuugcag....    254    0     seq
..........................................cuuucagucggauguuuAcag....    797    1     seq
..........................................cuuucagucggauguuuAcagc...    1819   1     seq
..........................................cuuucagucggauguuugcagc...    806    0     seq
..........................................cuuucagucggauguuugcagcu..    83     0     seq
...........................................uuucagucggauguuuA.......    1      1     seq
...........................................uuucagucggauguuugc......    17     0     seq
...........................................uuucagucggauguuuAc......    31     1     seq
...........................................uuucagucggauguuuAca.....    13     1     seq
...........................................uuucagucggauguuugca.....    1      0     seq
...........................................uuucagucggauguuugcag....    28     0     seq
...........................................uuucagucggauguuuAcag....    29     1     seq
...........................................uuucagucggauguuugcagc...    126    0     seq
...........................................uuucagucggauguuuAcagc...    123    1     seq
...........................................uuucagucggauguuugcagcu..    262    0     seq
...........................................uuucagucggauguuugcagcug.    11     0     seq
............................................uucagucggauguuuAcag....    1      1     seq
............................................uucagucggauguuuAcagc...    1      1     seq
```
